# Supplementary material for: Patterns of Intron Gain and Loss in Fungi
Source: PLoS Biol. 2004 Nov 30;2(12):e422. doi: 10.1371/journal.pbio.0020422 (PMC532390; doi:10.1371/journal.pbio.0020422)
Supplement: Table S1 — Also available at http://genes.mit.edu/NielsenEtAl/. (4.3 MB ZIP). [file pbio.0020422.st001.zip › NielsenEtAl/html/930.html]

AN4854.1.NCU04193.1.MG10057.1.FG04161.1


```
 CLUSTAL W (1.82) Multiple Sequence Alignments - Introns Inserted


Sequence 1: NCU04193.1	287 aa
Sequence 2: FG04161.1	272 aa
Sequence 3: MG10057.1	288 aa
Sequence 4: AN4854.1	269 aa
Alignment Length: 291 aa
Number Identitical Residues: 158 aa
Alignment Score (without introns) 7413


MG10057.1 	MAPTP--ETLASTVKLARSAALAAAPLAARHLMETSALHKRERPPGSLSVTDTQKVTLGV
NCU04193.1	MAPTSGSPSYHALVPSLDVSKRAALLVSTTTTLLTTTTTRRDLS----LQSDTQKITLGI
FG04161.1 	MAPASHSDTYSAAVEY--AAKRG-----------INALARRDLQ----NPNHTQQVTLGI
AN4854.1  	MAPYY------ALLTDRGIAAES-----------HSHLYKRDLT-----VNHTQAVTLGV
          	***        : :     :  .            .   :*:        ..** :***:

MG10057.1 	IAAYTVAIAILWNVPFLRWSLWPFK~MLVIAFHEFGHASTACCTGGRVESIKLDPREGGV
NCU04193.1	IFAYMLAIFILWNVPYIRSILWPFK~MLVIAFHEFGHAITCVLTGGRVLSISLDPREGGV
FG04161.1 	IGVYVVVIAILWNVPYVRMVLWPFK0MLVIAFHEFGHAITVLFTGGRVKSISLDPNEGGV
AN4854.1  	MAAYVVVIAILWNVPYLRYSLWPFK0MLVIAFHEFGHAITACCTGGRVKSISLDPHEGGV
          	: .* :.* ******::*  ***** ************ *   ***** **.***.****

MG10057.1 	TMMRGGISAITLPAGYLGSSLIGALLIFCGFDIIASKVVSFVLAVCFLLTLWWGKKDWLT
NCU04193.1	THMQGGRSGLTLPAGYLGSSLIGALLTFCGFDIVASKVASFVVGGCFLLTLWWGRRDWLT
FG04161.1 	THHIGGASAIVLPAGYLGSSIIGALLTFCGFNIVASKVASIVLGVCFLLTLWWGKRDWLT
AN4854.1  	THMQGGMAAITLPAGYLGSSIIGALLIFAGFNIIASKVASIVLGVCFLLTLWWARRDLLT
          	*   ** :.:.*********:***** *.**:*:****.*:*:. ********.::* **

MG10057.1 	IFTVLLAVGLLVACWFIAHAQALRFFV~LFIGVMSSLYSVWDI0CDDLILRKVNESDASQ
NCU04193.1	IGTILAAVGLLVGCWFIAHAEALRYVV~LFIGVMSSLYSVWDI0CDDLILRKVNSSDASV
FG04161.1 	ILTVLLAVGLLVACWFIKHAEALRFVV~LFIGVMSSLYSIWDI~CDDLILRKVNESDASV
AN4854.1  	IVTVLLAVGLLVACWFIAHGEALKWVV0LFIGVMSALYSVWDI0CDDLILRKVNSSDASV
          	* *:* ******.**** *.:**::.* *******:***:*** **********.**** 

MG10057.1 	FAKRYGGSSQCWGVIWSIISLAFMAAGIVAGIAAFPDSFAEQNEKSQRFLPTR-
NCU04193.1	FAKRYGGSSQCWGVIWSIISLGFMACGIIAGIAAFPQTRAQQQEDSQHFIPTKF
FG04161.1 	FAKRYGGSSQCWGVIWSIISILFMAAGIVAGLAVFSQSFEQQQKDSQKFIPT--
AN4854.1  	FAQRYGGSSRCWGLLWSLISLIFIAIAIVAAIAAFPQSFSQQEDDSKSFLPTKF
          	**:******:***::**:**: *:* .*:*.:*.*.::  :*:..*: *:**
```
